# Special Guest Editorial: Exploiting Complex Media Photonics to Illuminate Brain’s Hidden Depth

**DOI:** 10.1117/1.NPh.11.S1.S11501

**Published:** 2024-09-17

**Authors:** Hana Uhlířová, Tomáš Čižmár, Janelle M. P. Pakan, André Gomes

**Affiliations:** a Czech Academy of Sciences, Institute of Scientific Instruments, Brno, Czech Republic; b Leibniz Institute of Photonic Technology, Jena, Germany; c Institute of Applied Optics, Friedrich Schiller University Jena, Jena, Germany; d Otto-von-Guericke University, Magdeburg, Germany; e Leibniz Institute for Neurobiology, Magdeburg, Germany

## Abstract

The editorial introduces the Neurophotonics Special Issue “Complex Media NeuroPhotonics,” highlighting featured articles.

Understanding brain function is a captivating endeavor that has intrigued scientists and researchers for decades. Unravelling the mysteries of the brain necessitates a multifaceted approach, drawing knowledge from various fields far beyond traditional neuroscience.

In the last two decades, wavefront shaping and synthesis of structured light has been explored in virtually all aspects of photonics. In this special issue of *Neurophotonics*, we showcase technological advancements enabled by the field of complex media photonics, which strongly relies on this technological platform. Leveraging the algorithms, methodologies, and tools of wavefront shaping, accelerated by technological advances in digital modulators and detectors, gave birth to novel approaches addressing optical scattering — the fundamental limiting factor for high-resolution imaging deep into the brain. Complex media photonics has therefore brought about a great wave of optimism that wavefront shaping can overcome the degradation of light signals due to tissue scattering.

Amongst the prominent results of this endeavour are examples of technologies which have already reached translational maturity. For instance, the complex control of light in multimode and multicore fibers. These have versatile uses for imaging, including lens-less endoscopes, ultrathin endo-microscopes, and effective tethers for mini-scopes. The articles presented in this special issue highlight the transformative potential of these fiber-based technologies. This editorial casts a spotlight on several articles that feature in the special issue.

The article “Neurophotonics beyond the surface: unmasking the brain’s complexity exploiting optical scattering” (doi 10.1117/1.NPh.11.S1.S11510) gives a rich flavor of the complex media tools, outlining the opportunities and challenges in imaging the brain’s scattering tissues. “Fiber photometry-based investigation of brain function and dysfunction” (doi 10.1117/1.NPh.11.S1.S11502) underscores how depth-resolved fiber photometry can elucidate neural activities and their malfunctions.

Exciting developments have been achieved using multi-mode fibers (MMFs) as endoscopic probes for diffraction-limited resolution at unprecedented depths. Despite their rigidity constraint and the requirement for head-fixation in animal models, the potential for high-resolution imaging is immense, as highlighted in the perspective article “Fiber-based *in vivo* imaging: unveiling avenues for exploring mechanisms of synaptic plasticity and neuronal adaptations underlying behaviour” (doi 10.1117/1.NPh.11.S1.S11507).

On the other hand, fiber bundles offer great flexibility and can be utilized for lens-less imaging or as effective tethers for miniaturized microscopes. The advantages of using fiber bundles for all-optical brain studies in behaving animals are outlined in “Recent advances in light patterned optogenetic photostimulation in freely moving mice” (doi 10.1117/1.NPh.11.S1.S11508). In a different configuration, fiber bundles are used together with ultrathin photonic probes to achieve fast, high-resolution imaging in densely labelled brain regions, as discussed in “Photonic neural probe enabled microendoscopes for light-sheet light-field computational fluorescence brain imaging” (doi 10.1117/1.NPh.11.S1.S11503).

The transitional technology of multimode multi-core fibers combines the strengths of MMFs and fiber bundles, enabling high-resolution imaging in longitudinal studies of behaving animals.

The utility of computational approaches is exemplified in “Distinguishing motion artifacts during optical fiber-based *in-vivo* hemodynamics recordings from brain regions of freely moving rodents” (doi 10.1117/1.NPh.11.S1.S11511), which offers practical solutions to mitigate motion artifacts and enhance the reliability of *in-vivo* data. Furthermore, computational imaging and deep-learning methods applied to image reconstruction can further enhance imaging capacity toward higher resolution and demonstrate the broader potential of these fibers beyond neuroscience.

Finally, “Implantable photonic nano-modulators open perspectives for advanced optical interfaces with deep brain areas” (doi 10.1117/1.NPh.11.S1.S11512) introduces the concept of structuring fibers with nano-modulators, opening new avenues for interfacing with and modulating deep brain regions.

In conclusion, this special issue presents a rich collection of advancements in fiber-based brain imaging technologies. Each article contributes a vital piece to the overarching puzzle of understanding brain function, pushing the boundaries of what is achievable and paving the way for future breakthroughs in neuroscience. The integration of these sophisticated techniques holds the promise of unlocking deeper insights into the brain’s complexities, ultimately bringing us closer to decoding the secrets of this fascinating organ.

